# Heat Effects Are Unique: Mortality Risk Depends on Heat Wave, Community Characteristics

**DOI:** 10.1289/ehp.119-a81

**Published:** 2011-02

**Authors:** Tanya Tillett

**Affiliations:** **Tanya Tillett**, MA, of Durham, NC, is a staff writer/editor for *EHP*. She has been on the *EHP* staff since 2000 and has represented the journal at national and international conferences

During heat waves, higher-than-normal temperatures can present a deadly threat, with mortality occasionally doubling. Recent studies have demonstrated that heat-related mortality risk is influenced by the characteristics of the individual heat wave (such as heat intensity, duration, and timing in season). Researchers explored this relationship more fully in one of the largest multicity studies to date of heat wave impacts in the United States **[*EHP*** **119(2):210–218; Anderson and Bell]**.

The authors identified heat waves in 43 U.S. communities during the years 1987–2005. A heat wave was defined as 2 or more days in which temperatures exceeded the 95th percentile of warm season (May–September) temperatures for that community during the 19-year period. Each heat wave was characterized according to heat intensity (average mean temperature), duration in days, and the point in the season when the heat wave occurred.

The investigators estimated a 3.74% increase in average daily risk of nonaccidental death during the heat waves compared with non–heat wave days. Although longer and more intense heat waves were more common in the South, estimated effects of heat waves on mortality were greater in the Midwest and greatest of all in the Northeast. The authors attribute this phenomenon to Southern residents being perhaps more physiologically and behaviorally adapted to extreme temperatures. Nationwide, heat waves that occurred earlier in the warm season appeared to have a greater effect on mortality than heat waves occurring later (an average 5.04% increase compared with an average 2.65% increase), as did hotter or longer heat waves.

Considering that heat waves are expected to become more common and intense in some areas as the Earth’s climate changes, it is important to understand the factors that make individual communities vulnerable to heat-wave effects and that make individual heat waves more likely to cause excess deaths. The authors conclude it is important for officials to develop local response plans on the basis of heat-wave mortality trends in their own communities; when it comes to planning for health effects of heat waves, one size does not fit all.

## Figures and Tables

**Figure:**
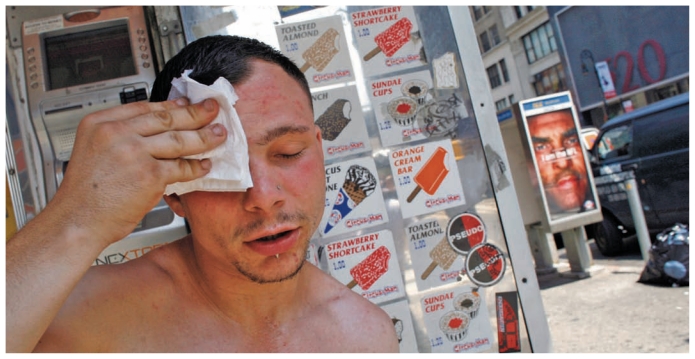
This Queens, New York, resident was photographed in the middle of a summer 2006 heat wave that ultimately would cause an 8% increase in nonaccidental deaths, including 40 heat-stroke deaths.

